# Doctors' Fees

**Published:** 1891-07-18

**Authors:** 


### Editor's Letter-Box.

[Our correspondents are reminded that prolixity is a great bar to publication, and that brevity of style and conciseness of statement greatly facilitate early insertion].

### DOCTORS' FEES.

I do not quite agree with the view expressed in the Annotation of July 4th regarding "doctors' fees." I think as a rule any amount is paid willingly and without questioning. It seems to me there are two subjects about which patients may reasonably complain. In the first place there are doctors who pay a great many unnecessary visits, thereby increasing their bill. But the greater evil is in the exorbitant fees which consulting physicians demand. Two or more guineas is large remuneration for a ten minutes' interview, and unfortunately there are people compelled to seek such an interview to whom even two guineas means a large sum, and spared with difficulty, and yet these people would be dismissed from a hospital as being too well off to receive medical charity. There should be some medium course to be adopted for people who are only quite middle class, as regards their position, but who are refined enough to dread the publicity and roughness that usually characterises hospital treatment. Why cannot every hospital have days on which, by paying moderately, patients whom I have described could be seen, and accorded privacy and civility, two conditions so essential to sensitive or nervous people?

Barnet.

